# A Microbiological Map of the Healthy Equine Gastrointestinal Tract

**DOI:** 10.1371/journal.pone.0166523

**Published:** 2016-11-15

**Authors:** Aaron C. Ericsson, Philip J. Johnson, Marco A. Lopes, Sonja C. Perry, Hannah R. Lanter

**Affiliations:** 1 University of Missouri Metagenomics Center, Department of Veterinary Pathobiology, University of Missouri, 4011 Discovery Drive, Columbia, MO, 65201, United States of America; 2 College of Veterinary Medicine, University of Missouri, 1600 Rollins Road, Columbia, MO, 65211, United States of America; 3 Department of Veterinary Medicine and Surgery, University of Missouri, 1600 Rollins Road, Columbia, MO, 65211, United States of America; Wageningen University, NETHERLANDS

## Abstract

Horses are exquisitely sensitive to non-specific gastrointestinal disturbances as well as systemic and extraintestinal conditions related to gut health, yet minimal data are available regarding the composition of the microbiota present in the equine stomach, small intestine, and cecum and their relation to fecal microbiota. Moreover, there is minimal information regarding the concordance of the luminal and mucosal microbial communities throughout the equine gut. Illumina-based 16S rRNA gene amplicon sequencing of the luminal and mucosal microbiota present in seven regions of the gastrointestinal tract of nine healthy adult horses revealed a distinct compositional divide between the small and large intestines. This disparity in composition was more pronounced within the luminal contents, but was also detected within mucosal populations. Moreover, the uniformity of the gut microbiota was much higher in the cecum and colon relative to that in the stomach, jejunum and ileum, despite a significantly higher number of unique sequences detected in the colon. Collectively, the current data suggest that while colonic samples (a proxy for feces) may provide a reasonable profile of the luminal contents of the healthy equine large intestine, they are not informative with regard to the contents of the stomach or small intestine. In contrast to the distinct difference between the highly variable upper gastrointestinal tract microbiota and relatively uniform large bowel microbiota present within the lumen, these data also demonstrate a regional continuity present in mucosal microbial communities throughout the length of the equine gut.

## Introduction

The equine gastrointestinal tract (GIT) is a remarkable organ system with a potential length and volume in the adult horse of over 30 meters and 150 liters, respectively. As in other mammals, the different anatomic regions of the equine GIT are specialized to perform specific functions, and each compartment must function correctly and in concert with the other regions to support the health of the animal. The stomach, divided into the non-glandular (dorsal) and glandular (antral or pyloric) regions, typically contains ingesta needed to buffer the abundant hydrochloric acid produced by gastric parietal cells. Some enzymatic digestion occurs in the stomach and, owing to the acid-tolerant bacteria that reside there [1], fermentation begins as well [2, 3]. The small intestines (i.e., duodenum, jejunum, and ileum), buffered to a near-neutral pH by pancreatic bicarbonate and hepatic bile, is the primary site of protein, soluble carbohydrate, and fat digestion and is also colonized by commensal microbial communities. Considering the rapid transit through the small intestine and relatively liquid content however, true colonization of the small intestine occurs primarily at the mucosal surface. Owing to its role in water resorption, the contents of the large intestine are more solid and contain the highest bacterial densities; it is also the primary sight of fermentation, resulting in the production of nutritionally beneficial short chain fatty acids (SCFAs) such as butyrate, propionate, and acetate. Reflecting the different functions of the various anatomic regions, there are also differences in the rate of throughput [4], pH [5], available energy sources, and epithelial architecture of each region.

In addition to differences in microbial composition between compartments of the equine GIT, differences between the luminal and mucosal communities is also of clinical interest. There are different means by which gut microbes can influence host physiology, including contact-dependent and independent mechanisms. Recognition of the autochthonous microbiota and response by the host immune system requires direct contact, be it via M cell-mediated transcytosis or fractalkine-mediated sensing by dendritic cells [6]. In contrast, many metabolites such as SCFAs and secondary bile acids produced by luminal microbes not in direct contact with the epithelium have been shown to confer beneficial and detrimental effects, respectively [7]. Thus, differences between compartments in the mucosal communities could influence pathways mediated through direct contact mechanisms while differences in luminal populations could be of greater clinical relevance to those pathways responding to microbial metabolites produced in the gut lumen.

With the aforementioned differences in mind, the utility of fecal samples as a representative sample of the gut microbiota is problematic. Considering both the exquisite sensitivity of horses to gastrointestinal health and the high prevalence of vague diagnoses such as “colic” and “microbial dysbiosis” in the clinical setting, it is critical to gain a more comprehensive understanding of the microbial populations colonizing the GIT of healthy horses, in order to differentiate abnormal microbial compositions. To generate a detailed profile of the healthy gut microbiota, luminal contents and mucosal scrapings were collected from several regions throughout the GIT of adult horses subjected to euthanasia for reasons unrelated to enteric health and subjected to culture-independent characterization via 16S rRNA gene amplicon sequencing. Bioinformatics and statistical analysis were used to identify and visualize differences between the microbiota present in different anatomic regions, as well as between luminal and mucosal microbial communities.

## Materials and Methods

### Horses and sample collection

All samples were obtained from horses subjected to euthanasia at the University of Missouri Veterinary Medical Teaching Hospital for reasons unrelated to gastrointestinal conditions, during the months of April and May of 2015. Additionally, all horses were subjected to euthanasia for reasons unrelated to the current study. As such, all samples were collected *post mortem* and, per the University of Missouri, Institutional Animal Care and Use Committee (IACUC), no IACUC approval was needed. Prior to euthanasia, horses underwent a physical examination by a veterinary internal medicine specialist (equine), and were deemed to be free of overt metabolic or intestinal disease. Owners were specifically queried regarding medical treatments in the period preceding sample collection; no horses had received antibiotics or anti-inflammatory medications in the three months preceding euthanasia. Horses enrolled in the present study were of multiple breeds and ages, and included both females and castrated males; **Table 1**below provides details regarding the demographics.

**Table 1:** Demographic data associated with horses included in the study.

#	Date of euthanasia	Age	Breed	Sex	Condition	Reason for euthanasia
**1**	24 April 15	6	Morgan	MC	WNL	Old wound
**2**	4 May 15	10	Quarter Horse	F	WNL	UHPH
**3**	5 May 15	12	Quarter Horse	F	WNL	UHPH
**4**	6 May 15	11	Quarter Horse	F	WNL	UHPH
**5**	7 May 15	15	Quarter Horse	MC	WNL	UHPH
**6**	15 June 15	10	Quarter Horse	F	WNL	Untrainable
**7**	17 June 15	8	Paint	MC	WNL	Untrainable
**8**	18 June 15	17	Quarter Horse	F	WNL	Untrainable
**9**	19 June 15	15	TWH	MC	WNL	Navicular disease

TWH, Tennessee Walking Horse; WNL, within normal limits; UHPH, unwanted healthy pasture horse

Following euthanasia, performed according to the 2013 Guidelines for the Euthanasia of Animals, horses were examined grossly *post mortem* for signs of intestinal or systemic disease. Full-thickness samples of the gastrointestinal tract measuring approximately 25 cm^2^ were excised from the following locations: dorsal (squamous epithelial) stomach, antral (glandular epithelial) stomach, jejunum, ileum, cecum, ventral (ascending) colon, and dorsal (descending) colon. Care was taken to assure that gastrointestinal sample locations were similar between horses. Representative luminal (digesta) samples were also obtained from the same sampled locations, with dorsal colonic samples serving as a surrogate for feces. All samples were collected and placed on ice within 30 minutes of euthanasia. Upon collection, luminal contents were placed in 50 mL conical tubes and stored at -80°C until DNA extraction was performed. For mucosal samples, excised tissue from each region of the GIT was thoroughly rinsed with sterile saline until all grossly visible digesta had been removed, and then stored at -80°C until DNA extraction was performed. Histopathological examination of all gastrointestinal segments of interest was undertaken to rule out morphologic changes in tissue architecture related to disease.

### DNA extraction

For extraction of luminal contents, a small portion was removed from the frozen sample immediately prior to DNA extraction and the remaining sample was returned to the freezer. For extraction of mucosal DNA, tissue sections were removed from the freezer and the epithelial surface was scraped vigorously with a sterile scalpel blade to collect material for extraction. DNA was extracted using MoBio PowerFecal kits (Carlsbad, CA), according to the manufacturer’s recommendations, with minor adaptations due to equipment availability. Briefly, 0.25 mg of luminal contents or material collected via mucosal scrape was added to the dry bead tube containing 750 μL of bead solution and gently vortexed. C1 solution was added, the sample briefly vortexed, and incubated at 65°C for 10 minutes following the recommended protocol. Samples were shaken for 10 min. in a TissueLyser II (Qiagen, Venlo, Netherlands) at 30Hz. Samples were centrifuged at 13,000 × g for 1 min., the supernatant transferred to the provided 2 mL collection tube, and the remainder of the protocol was followed as recommended by the manufacturer. All samples were eluted in 100 μL solution C6.

### Library construction and sequencing

16S rRNA gene amplicon library construction and sequencing were performed at the University of Missouri DNA Core facility. DNA concentration of samples was determined fluorometrically and all samples were normalized to a standard concentration for PCR amplification. Bacterial/archaeal 16S rRNA gene amplicons were generated via amplification of the V4 hypervariable region of the 16S rRNA gene using single-indexed universal primers (U515F/806R)[8] flanked by Illumina standard adapter sequences and the following parameters: 98°C^(3:00)^+[98°C^(0:15)^+50°C^(0:30)^+72°C^(0:30)^] × 25 cycles +72°C^(7:00)^. Amplicons were then pooled for sequencing using the Illumina MiSeq platform and V2 chemistry with 2×250 bp paired-end reads, as previously described [9]. Samples returning greater than 1000 reads were included in the subsequent analyses. **S1 Table** shows the range, mean, median, and SD in coverage by sample site.

### Informatics analysis

All informatics analysis was performed as previously described [10], at the MU Informatics Research Core Facility. Briefly, assembly of DNA contigs was performed using FLASH software [11], and culled if found to be short after trimming for a base quality less than 31. *De novo* and reference-based chimera detection and removal was performed using Qiime v1.8 [12] software, and remaining contiguous sequences were assigned to operational taxonomic units (OTUs) via *de novo* OTU clustering and a criterion of 97% nucleotide identity. Annotation of selected OTUs was performed using BLAST [13] against the Greengenes database [14] of 16S rRNA gene sequences and taxonomy. Principal component analyses were performed using ¼ root-transformed OTU relative abundance data via a non-linear iterative partial least squares algorithm, using an open access Excel macro available from the Riken Institute [15], downloaded on August 10, 2015. Hierarchical clustering was performed via the unweighted pair group method with arithmetic mean (UPGMA) and Bray-Curtis similarity indices, using the Past 3.12 software package [16], downloaded on April 2, 2016.

### Statistical analysis

Sequence and OTU richness and diversity indices were first tested for normality using the Shapiro-Wilk method; differences were then tested via one-way ANOVA or Kruskal-Wallis ANOVA on ranks for normal and non-normal data respectively using SigmaPlot 12.3 (Systat Software Inc., San Jose, CA); *p* values less than 0.05 were considered significant. Differences in the overall composition of the different regions (stratified between luminal and mucosal samples) were tested via one-way PERMANOVA of ranked Bray-Curtis similarity indices using the open access Past 3.12 software package [16].

## Results

**Fig 1:**
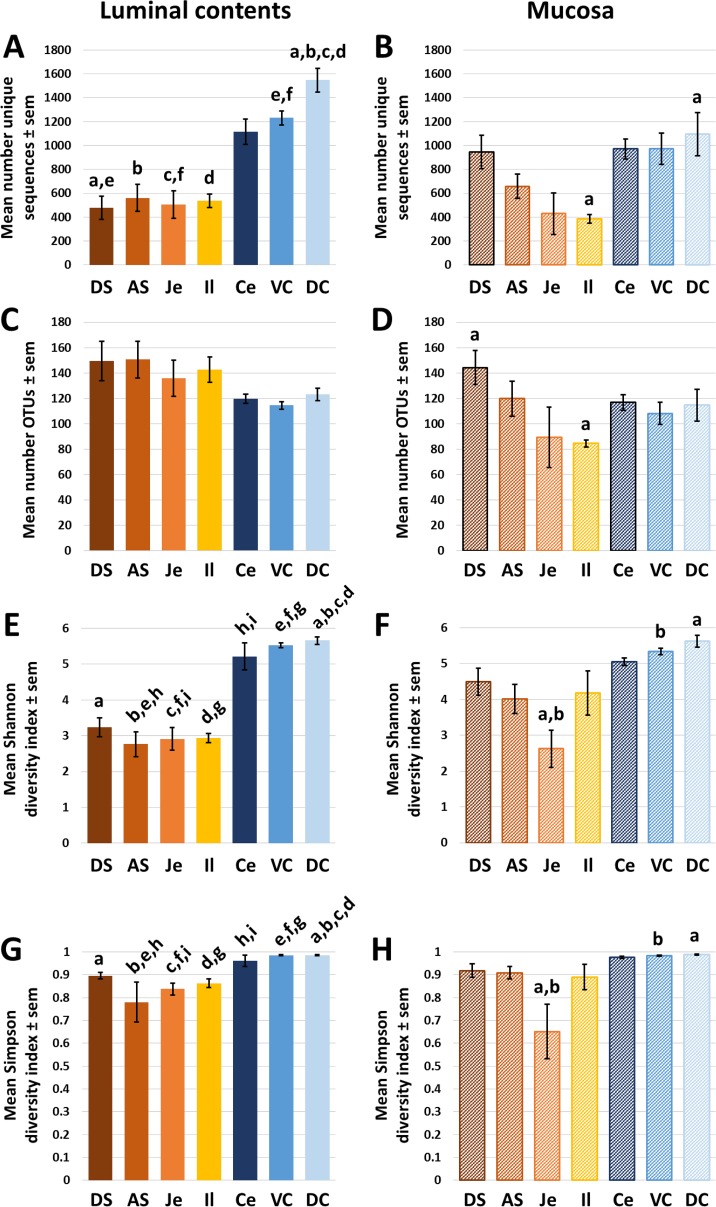
Richness of the luminal and mucosal equine gut microbiota. Bar charts showing the mean (± sem) number of unique 16S rRNA gene amplicon sequences (**A**,**B**) or operational taxonomic units (OTUs; **C**,**D**) detected in luminal contents (**A**,**C**) or mucosa (**B**,**D**) of samples collected from dorsal stomach (DS), antral stomach (AS), jejunum (Je), ileum (Il), cecum (Ce), ventral colon (VC), or dorsal colon (DC) of nine healthy adult horses. Bars within a chart marked with like letters are significantly different (*p* < 0.05, ANOVA).

To determine if there were differences in the overall richness of microbial populations within the lumen, or adherent to the mucosa, of different regions of the gut, the number of total unique (i.e., distinct from all other sequences at a minimum of one base pair) sequences detected was compared. Within the lumen of the GIT, there was a trend, albeit statistically insignificant, toward greater numbers of unique sequences being detected in the large intestine relative to the stomach and small intestine. Differences between the ventral or dorsal colon and the stomach and small intestinal samples achieved statistical significance in several comparisons (**Fig 1A**). Comparisons of the richness (i.e., the total number of unique sequences, regardless of distribution) of mucosal populations revealed a similar pattern although the apparent richness was not as uniform across the gastric and small intestinal samples. Rather, the number of unique sequences recovered from the dorsal stomach samples was comparable to that detected in the large intestinal samples, and the richness of mucosal populations declined gradually throughout the next several anatomic regions until increasing abruptly in the large intestine (**Fig 1B**). While the number of unique amplicon sequences is one commonly used metric of microbial richness, many of these sequences may differ only slightly between closely related taxa. To account for this, the numbers of total operational taxonomic units (OTUs), or clusters of sequences sharing at least 97% nucleotide identity, were also compared. Interestingly, the trend toward increased richness in the luminal contents of the large intestines was reversed; greater numbers of OTUs were detected in the gastric and small intestinal luminal samples relative to the cecal and colonic samples, although these differences did not reach statistical significance (**Fig 1C**). The pattern in number of OTUs detected in mucosal samples collected from different regions of the GIT was similar to the comparison of unique sequences (**Fig 1D**). Collectively, these data indicate that, while the true richness of the microbial communities within the lumen are relatively low and stable within the upper GIT and then increase in the lower GIT, the phylogenetic relatedness of microbiota is greater in the lower GIT, suggesting environmental pressures selecting a subset of more closely related microbes in the lumen of the large intestine. Conversely, the richness of mucosal populations followed a similar pattern regardless of whether the total number of unique sequences or OTUs was compared. To account for the distribution of the detected taxa, Shannon (**Fig 1E** and **Fig 1F**) and Simpson (**Fig 1G** and **Fig 1H**) α-diversity indices were also determined. These indices are a function of both richness and evenness, with greater α-diversity indicating a more even distribution. Notably, analysis via ANOVA with post hoc pairwise comparisons detected multiple differences in both indices between the luminal contents in regions of the upper and lower GIT. Alternatively, there were few differences in α-diversity detected between mucosal samples from different segments of the GIT, the exception being lower α-diversity in the jejunal mucosa as compared to the ventral and dorsal colonic mucosa.

**Fig 2:**
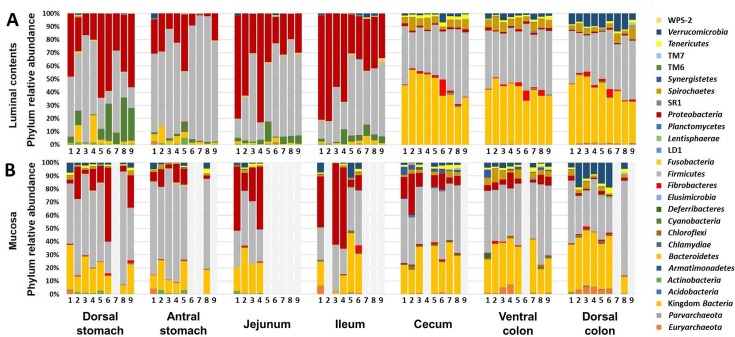
Relative abundance of phyla in luminal and mucosal equine gut microbiota. Bar charts showing relative abundance of phyla detected in luminal contents (**A**) and mucosa (**B**) of samples collected from dorsal stomach, antral stomach, jejunum, ileum, cecum, ventral colon, and dorsal colon of nine healthy adult horses, displayed in the same order in each chart and in each GIT region (animal IDs listed below bars). Samples returning fewer than 1000 sequences not shown; legend at right.

To assess similarities and differences in community structure, stacked bar charts showing the relative abundance of phyla were generated. Of the 27 different phyla detected across the entire set of samples, the luminal contents of the upper GIT contained high proportions of bacteria in the phyla *Proteobacteria* and *Firmicutes*, lower relative abundance of *Cyanobacteria* and *Bacteroidetes*, and much lower relative abundance of the remaining 23 phyla (**Fig 2A**). The composition of luminal contents changed substantially at the junction between the small and large intestines to one dominated by *Firmicutes* and *Bacteroidetes*. The phyla *Verrucomicrobia*, *Tenericutes*, *Spirochaetes*, and *Fibrobacteres* were detected at lower, but appreciable, relative abundances in the cecal and colonic lumen of all horses. Subjectively, there was also greater variability in the gastric and small intestinal samples relative to the cecal and colonic samples. Interestingly, the division in community composition between upper and lower GIT was much less evident in mucosal samples. With the exception of *Cyanobacteria*, all of the aforementioned phyla detected in the luminal contents were consistently detected on the mucosa of all anatomic regions (**Fig 2B**). While the relative abundance of several phyla shifted between regions of mucosa, those changes occurred somewhat gradually along the length of the GIT and the transition from upper to lower GIT was much less distinct.

**Fig 3:**
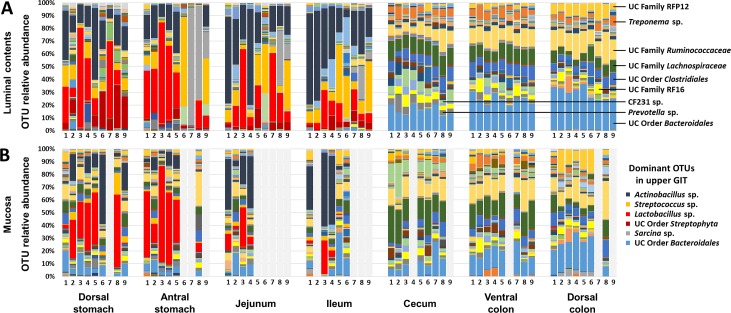
Relative abundance of operational taxonomic units in luminal and mucosal equine gut microbiota. Bar charts showing relative abundance of operational taxonomic units (OTUs) detected in luminal contents (**A**) and mucosa (**B**) of samples collected from dorsal stomach, antral stomach, jejunum, ileum, cecum, ventral colon, and dorsal colon of nine healthy adult horses, displayed in the same order in each chart and in each GIT region (animal IDs listed below bars). Samples returning fewer than 1000 sequences not shown; dominant OTUs in upper gastrointestinal tract (GIT) labeled at upper right, legend for dominant OTUs in lower GIT at lower right.

Resolved to the level of OTU, the luminal contents demonstrated a similar pattern. There was an apparently abrupt change in the composition of luminal contents at the transition from small to large intestines, and a more uniform composition in the cecal and colonic communities (**Fig 3A**). The most abundant taxa detected in the upper GIT included *Lactobacillus* sp., *Streptococcus* sp., *Actinobacillus* sp., *Sarcina* sp., and unclassified (UC) bacteria in the family *Enterobacteriaceae* (i.e., UC family *Enterobacteriaceae*) and UC order *Streptophyta*. Alternatively, the composition was much less variable between horses within the lumen of the cecum, and ventral and dorsal colon. Additionally, while certain OTUs accounted for very large proportions of the detected DNA within regions of the upper GIT, no single OTU made up more than 35% of the overall DNA recovered from any lower GIT sample. Specifically, the most abundant taxa detected in the cecum and colon included UC order *Bacteroidales*, *Prevotella* sp. (primarily cecum and ventral colon), UC family RF16, genus CF231, UC order *Clostridiales*, UC families *Lachnospiraceae* and *Ruminococcaceae*, *Treponema* sp., and UC family RFP12.

In contrast to the annotations at the phylum level, when resolved to the level of OTU, the mucosal populations in the stomach and small intestines appeared similar to the luminal contents of those regions, and very distinct from the mucosal populations detected in the cecum and colon (**Fig 3B**). Specifically, *Lactobacillus* sp. represented a dominant taxon accounting for up to 74% of the DNA isolated from the antral stomach of one horse. Similarly, *Actinobacillus* sp. was also found in high relative abundance of several mucosal samples from the upper GIT, similar to the luminal contents. The composition of cecal and colonic mucosal communities, at the OTU level, was very similar to those found in the lumen, with some exceptions. One clear difference was a subjectively greater relative abundance of *Desulfovibrio* sp. on the cecal mucosa, compensated for by a reduction in the proportion of *Treponema* sp. and unclassified microbes in the UC order *Clostridiales*. In the ventral and dorsal colonic mucosa, a substantial proportion of DNA was annotated to the archaea *Methanobrevibacter* sp. and *Methanocorpusculum* sp. A full list of all OTUs detected at greater than 0.01% mean relative abundance in the luminal or mucosal samples of at least one region of the GIT are included in **S2 Table** and **S3 Table**, respectively.

**Fig 4:**
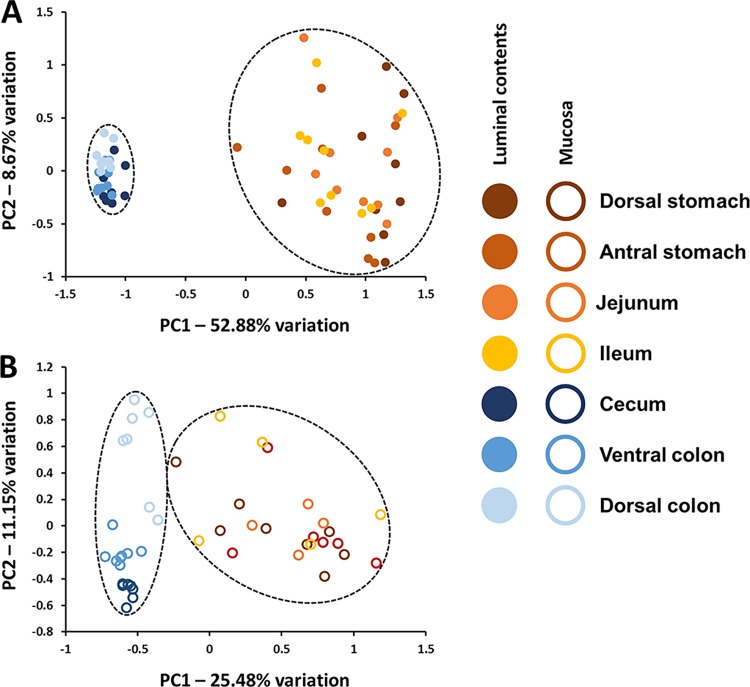
Separate principal component analysis of luminal and mucosal gut microbial populations. Principal component analysis of microbial communities detected in luminal contents (**A**) and mucosa (**B**) of samples collected from dorsal stomach, antral stomach, jejunum, ileum, cecum, ventral colon, and dorsal colon of nine healthy adult horses; legend at right.

While visual inspection of bar charts allows for identification and comparison of prominent taxa, it is difficult to appreciate differences in the presence or relative abundance or rare OTUs. In order to better assess compositional differences between samples, taking into account all detected OTUs, principal component analysis was performed. Comparing first the luminal contents of the various anatomic regions, samples formed two distinct clusters separated along principal component 1 (PC1, 52.88% variation) representing upper and lower GIT respectively (**Fig 4A**). There was considerable β-diversity among the upper GIT samples along both PC1 and PC2, while samples from the lower GIT clustered very tightly, indicating relatively high compositional similarity between cecal and colonic samples, as well as between horses. Agglomerative hierarchical clustering was performed as a secondary evaluation of β-diversity (S1 Fig). Validating the PCA, samples from the upper and lower GIT formed two distinct branches of the dendrogram, as did samples from the dorsal colon. Statistical testing of overall community differences between the luminal contents via PERMANOVA confirmed a significant main effect of gut region (*p* = 0.0001; F = 16.45); pairwise comparisons indicated that while the microbiota of the upper GIT did not differ between regions, the microbiota present in each region of the lower GIT lumen was significantly different from all other regions (including other regions of the lower GIT (**Table 2**).

**Table 2:** Pairwise PERMANOVA of Bray-Curtis similarity indices between luminal samples.

	DS	AS	Jejunum	Ileum	Cecum	VC	DC
DS		1	1	**0.0084**	**0.0021**	**0.0042**	**0.0042**
AS	2.1		1	0.1176	**0.0042**	**0.0021**	**0.0063**
Jejunum	1.42	1.255		1	**0.0021**	**0.0021**	**0.0042**
Ileum	3.26	3.079	0.5826		**0.0021**	**0.0021**	**0.0021**
Cecum	31.12	19.1	29.39	37.64		**0.0042**	**0.0021**
VC	34.01	20.42	31.71	41.21	4.137		**0.0021**
DC	33.58	20.22	30.82	40.06	15.82	6.917	

Results of one-way PERMANOVA testing of ranked Bray-Curtis similarity indices between luminal microbiota samples in sequential regions of the equine gastrointestinal tract. Bonferroni-corrected *p* values of pairwise comparisons shown in upper right with significant differences depicted in bold; F values shown in lower left. DS = dorsal stomach, AS = antral stomach, VC = ventral colon, DC = dorsal colon.

Regarding the mucosal samples, a similar pattern was observed although the separation between upper and lower GIT was not as strong, with PC1 explaining 25.48% variation (**Fig 4B**). Notably, mucosal samples collected from the cecum and two colonic regions separated completely along PC2 (11.15% variation), indicating that there are region-specific features of the mucosal microbiota that were not readily apparent in the luminal contents. Hierarchical clustering reflected a similar relationship between samples with two main branches comprising primarily upper and lower GIT samples respectively, although the separation was not as absolute as with the luminal samples (S2 Fig). Again, testing via PERMANOVA detected a significant effect of region on the microbiota (*p* = 0.0001; F = 7.277), and significant differences in the composition of the mucosal samples between certain regions of the upper and lower GIT although those differences were not as complete as in the luminal samples (**Table 3**).

**Table 3:** Pairwise PERMANOVA of Bray-Curtis similarity indices between mucosal samples.

	DS	AS	Jejunum	Ileum	Cecum	VC	DC
DS		1	**0.0336**	1	**0.0084**	**0.0021**	**0.0189**
AS	0.9533		0.2016	0.9786	**0.0126**	**0.0105**	**0.0105**
Jejunum	7.222	5.142		0.1743	0.0588	**0.0483**	0.0588
Ileum	1.363	2.358	3.508		**0.0147**	**0.0063**	0.1029
Cecum	9.03	10.02	17.3	6.567		0.0651	**0.021**
VC	10	12.01	22.8	7.332	3.773		**0.0273**
DC	8.066	9.514	16.58	4.916	9.083	5.758	

Results of one-way PERMANOVA testing of ranked Bray-Curtis similarity indices between mucosal microbiota samples in sequential regions of the equine gastrointestinal tract. Bonferroni-corrected *p* values of pairwise comparisons shown in upper right with significant differences depicted in bold; F values shown in lower left. DS = dorsal stomach, AS = antral stomach, VC = ventral colon, DC = dorsal colon.

**Fig 5:**
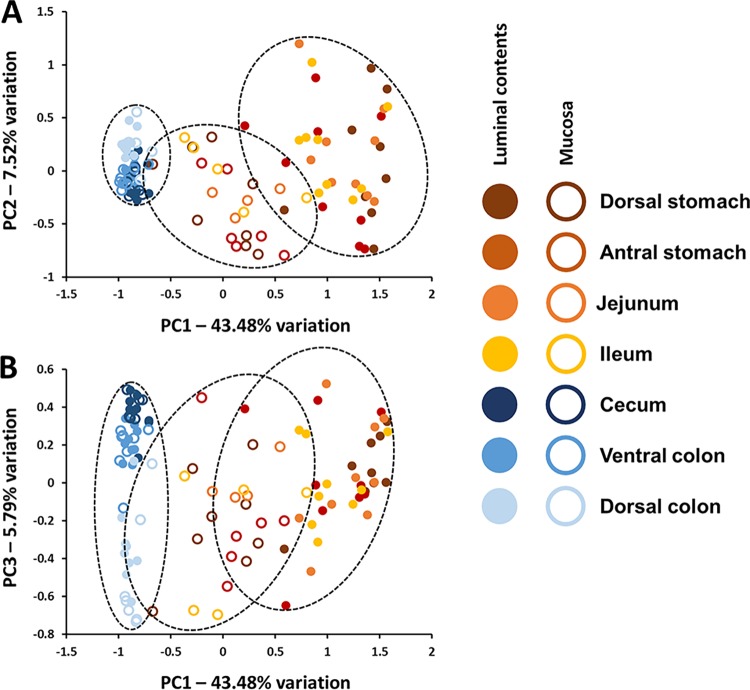
Combined principal component analysis of luminal and mucosal gut microbial populations. Principal component analysis of microbial communities detected in luminal contents and mucosa of samples collected from dorsal stomach, antral stomach, jejunum, ileum, cecum, ventral colon, and dorsal colon of nine healthy adult horses, shown as PC1 versus PC2 (**A**) and PC1 versus PC3 (**B**); legend at right.

To evaluate compositional differences between luminal and mucosal microbial communities, PCA was also performed using all samples. While the luminal and mucosal samples collected from the upper GIT formed two distinct but slightly overlapping clusters when viewed as PC1 versus PC2, all of the samples from the lower GIT were contained in a single small cluster (**Fig 5A**). Mucosal samples from the upper GIT were situated on PC1 midway between luminal samples from the same anatomic regions and the cluster of samples from the lower GIT. Viewed as PC1 versus PC3, the lower GIT samples again separated by anatomic region with minimal discrimination between luminal and mucosal samples (**Fig 5B**). Collectively, the PCAs above suggest that the composition of microbial communities within the stomach, jejunum, and ileum are distinct from those in the cecum and colon, and much more variable between horses. Additionally, while there was no clear region-specific separation of either luminal or mucosal samples from the upper GIT, samples collected from the lower GIT separated according to anatomic region suggesting subtle but consistent differences between the microbial populations colonizing both the lumen and mucosa of the cecum, ventral colon, and dorsal colon. Taking into account all samples, two-way PERMANOVA of ranked Bray-Curtis similarity indices confirmed significant main effects of anatomic region (e.g., dorsal stomach, antral stomach, *p* = 0.0001; F = 12.174) and sample collection (i.e., lumen versus mucosa, *p* = 0.0001; F = 118.7), as well as a significant interaction between fixed variables (*p* = 0.0001; F = 4.9469).

## Discussion

The equine industry contributes roughly $39 billion in direct impact to the U.S. economy annually, and an additional $60 billion in indirect or induced impact. Individually, horses are among the most expensive companion animals to feed and maintain and, considering their potential longevity, the lifetime cost of horse ownership easily exceeds that of all other companion species. In horses, non-specific abdominal pain or “colic” is one of the most common causes of morbidity and mortality, with an estimated incidence of 4.2 events per horse per year [17]. Case fatality rates vary among studies but have been calculated to be between 7% and 13% [17–19]. Considering the high incidence of these conditions in conjunction with the considerable expense of treating horses, the total annual economic impact of equine colic in the U.S. was estimated to be over $115 million in 1998 and has surely increased since then [17]. It should be noted that despite the term “colic”, signs of abdominal pain can originate from other regions of the gut or other organ systems. Moreover, no etiology is determined in the majority of cases [19, 20], although the most common clinical associations include gas colic and feed-related issues [17, 21]. It is plausible that in many of the idiopathic cases, the underlying cause of discomfort, bloating, ileus, or other clinical signs may be related to changes in the composition or function of the resident microbiota. Moreover, several other common disease entities affecting the musculoskeletal [22, 23] and metabolic system [24] of horses are also intimately associated with the gastrointestinal microbiota. The majority of intestinal microbes are refractory to culture and the ability to interrogate these rich and dynamic microbial communities using culture-independent methods has only recently become technically and economically feasible. Indeed, several groups have now described the composition of fecal [25–31] and gastric [32] microbiota in healthy horses, as well as associations between specific conditions affecting horses (such as laminitis [22], colic [31], and colitis [33]) and characteristic changes in the fecal microbiota. That said, there are still many uncertainties regarding the healthy equine gut microbiota including the composition of the gastric and small intestinal microbiota in relation to that colonizing the cecum and colon, and the composition of mucosal communities in relation to the luminal contents. For the purposes of diagnosing and treating upper GI disturbances related to the gut microbiota (GM), these differences may very well be of clinical relevance.

The present data agree with previous findings [30] in that, with regard to luminal microbial populations, there are two distinct regions of the equine gastrointestinal tract. The microbial composition within the upper GIT, represented here by the stomach, jejunum, and ileum, was highly variable between horses, and between regions within the same horse. While many of the same dominant taxa (e.g., *Actinobacillus* sp. and *Lactobacillus* sp.) were detected in these regions across all horses, the relative abundance varied substantially. This likely reflects both the relatively high rate of throughput in the upper GIT, and the constant introduction of environmental bacteria present in the feed and forage. Supporting this, the upper GIT contained abundant α-*Proteobacteria*, common in environmental samples. Conversely, the luminal contents of the lower GIT, i.e., the cecum and colon, were remarkably similar between horses despite the variable history, breed, and age of the horses included in the study. It merits emphasis that, despite the lack of uniformity within the lumen of regions of the upper GIT, the luminal and mucosal populations of the lower GIT were remarkably uniform, suggesting that the cecal and colonic microbial populations are stable and affected only minimally by differences higher in the GIT. Due to logistical constraints, all horses spent at least 18 hours in the University of Missouri, Veterinary Medical Teaching Hospital equine clinic prior to euthanasia. Thus, while some normalization in the composition of the GM may have occurred owing to the common environment preceding sample collection as well as the fact that all horses resided in rural Missouri, it is doubtful that the common feed and housing conditions for such a short period of time could induce such a uniform microbial composition in horses of multiple ages, breeds, and backgrounds.

The data presented here are also supported by their agreement with previous analyses of the equine gut microbiota performed using 16S rRNA gene sequencing methods, although there are also differences worthy of mention. Several studies applying next-generation sequencing methods to healthy equine hindgut samples have detected *Firmicutes*-dominated microbial profiles, followed in relative abundance by some combination of *Bacteroidetes*, *Proteobacteria*, or *Verrucomicrobia* as the second and third most prevalent phyla, depending on the study [22, 26, 31, 33]. In all of these studies, bacteria in the phylum *Firmicutes* outweighed the next most common phylum by a ratio of 4:1 or greater, in contrast to the present data wherein the *Firmicutes* to *Bacteroidetes* ratio was approximately 1:1 in all horses. These discrepancies can be explained by several possible factors including geographic location, breed, and husbandry of the horses, as well as methodological differences such as DNA extraction technique and sequencing platform. It is doubtful that this difference in sequencing platform accounts for the difference in detected phyla as one group has utilized both systems [29, 30, 33, 34] with samples collected from horses in a similar environment, and generated comparable results. Of note however, studies performed by Dougal et al. [27, 28], using samples obtained from different groups of horses located in Michigan and the UK respectively, detected a *Firmicutes* to *Bacteroidetes* ratio similar to that reported here. Thus, the differences in detected *Firmicutes* to *Bacteroidetes* ratio may be a function of geographic location, thus indicating a substantial range of values in the healthy equine hindgut. This is further supported by comparison of the current data to that generated in the similarly designed study performed in Ontario, Canada. While the extraction chemistry and primer set varied slightly from those used here, sequencing of the same 16S rRNA gene region detected a *Firmicutes*-dominated hindgut[30]. Alternatively, such variance may be attributable to seasonal effects on forage and thus the GM.

Considering the proposed concept of a “core” fecal microbiota in horses, i.e., a group of OTUs that are detected at greater than 0.1% relative abundance in all [27] or a majority [35] of samples, 87 and 98 OTUs were detected at greater than 0.1% mean relative abundance in the lumen and mucosa of the dorsal colon respectively (a surrogate for feces). Of those, 68 and 42 OTUs respectively were detected in all nine samples, 41 of those being shared between the luminal and mucosal samples. Comparison of these numbers to existing literature however is problematic as they are dependent on sequencing coverage and the resolution afforded by the primer set.

Progressing to more proximal regions of the GIT, the cecum appears very similar compositionally to the ventral and dorsal colon, although the relative abundance of certain OTUs shifts throughout the length of the GIT. Conversely, the ileum appears in stark contrast to the hindgut, suggesting a dramatic shift in the community architecture. Moreover, the agreement between horses is much less in the upper GIT, as evidenced by the distribution of those samples on PCA. These data are in agreement with the findings of Dougal et al. who found that, while samples from the cecum, various colonic regions, and feces were intermingled in a hierarchical clustering analysis, ileal samples formed a separate branch of the dendrogram [27]. Thus, the ileocecal junction represents a transition zone within the equine GIT, leading to a more uniform microbial community. The reason for this stark shift in the composition and uniformity of the luminal microbiota is unclear but is likely due, at least in part, to the change in throughput as ingesta reach the hindgut. In the foregut, transit is relatively rapid whereas in the hindut, transit slows considerably. Additionally, there are surely differences in substrate availability between the upper and lower GIT; as most dietary macromolecules are absorbed in the jejunum, the bacteria present in the hindgut become more reliant on fermentation of non-digestible carbohydrates. Regarding the greater variability between horses in the composition of the upper GIT microbiota, it must also be considered that an occult condition, undetected during the physical, gross, and histological examinations, influenced the results. While the screening criteria for inclusion was intended to eliminate horse with gastrointestinal disease, certain sub-clinical conditions could ostensibly affect the GM while being undetected during physical and histological examination to screen subjects.

Regarding the equine gastric microbiota, there is limited available literature describing culture-independent studies of the equine gastric microbiota. Yuki et al. performed an informative analysis of the *Lactobacillus* spp. colonizing the epithelium of the equine stomach, however isolates were first selected via culture methods [1]. Subsequent studies using biopsy material collected from the stomach of six healthy horses and subjected to 16S rRNA gene pyrosequencing revealed a rich microbiota comprising many more taxa than *Lactobacillus* spp. [32] Supporting the current data set, that study identified no apparent microbial differences between the glandular and nonglandular regions of the stomach.

There are, of course, limitations to the present study including the relatively small sample size and the inability to control for multiple variables of potential import such as diet, sex, age, and activity level. While all of these variables have been documented to influence the composition of GM in humans [36–40], little is known regarding the effect of these variables in equine samples. Willing et al. used terminal-restriction fragment length polymorphism analysis in combination with sequencing to demonstrate the energy-rich forage diets led to decreased abundance of lactic acid bacteria and certain *Streptococcus* spp. [25] but, to the authors’ knowledge, there are no comparable published data generated with next-generation sequencing. Similarly, in the current study and the previous work of Costa et al. [30] wherein horses of both sexes were analyzed, no sex-dependent differences were noted.

Another limitation to the present study, as well as the referenced surveys of the equine GM, is the limited resolution and relative abundance of taxa provided by 16S rRNA gene amplicon sequencing. In contrast to whole genome shotgun sequencing, targeted sequencing of 16S rRNA gene libraries can only discriminate microbial taxa down to the level of species but most sequences are annotated to only the level of genus or family. Thus, while robust changes in the composition of polymicrobial communities may be detected with excellent reproducibility, changes within certain unresolved taxa could go undetected. Additionally, both targeted and whole genome sequencing methods report the relative abundance of detected taxa and absolute quantification of any given taxa requires different methodologies.

Lastly, while the material collected from any given segment of the GIT appeared relatively homogenous, it is possible that the 250 mg collected for analysis may not be representative of the region. The multiple technical replicates needed to answer those questions would have been prohibitively expensive and, based on the inter-subject agreement between those regions harboring the highest microbial densities, of limited value. Similarly, the data reported herein represent a relatively low sample size, particularly in the upper GIT mucosa, owing to the failure of several samples to amplify and sequence well. Thus, this relatively low sample size may have reduced our ability to detect differences between regions in the upper GIT, particularly the jejunum.

While the above data provide a detailed profile of the microbial populations present throughout the length of the equine GIT, additional studies are needed to determine the effect of geographical and seasonal differences in sample collection. Moreover, the associations between various conditions (e.g., colic, laminitis, and obesity/insulin dysregulation) and characteristic shifts in the composition of the equine GM need to be reproduced and examined for causality. Approaches such as longitudinal fecal sampling of horses at an increased risk of such conditions, or fecal bacteriotherapy of affected horses with microbial samples collected from healthy donors would begin to answer those questions. Once any such causal relationships are found, targeted analysis of the metabolome or proteome could be used to develop improved diagnostic and therapeutic approaches.

Collectively, the data presented above suggest that while fecal samples may provide a reasonable profile of the luminal contents of the equine large intestine, the dorsal colon microbiota is nonetheless significantly different from that present in the cecum and ventral colon, and is entirely uninformative with regard to the contents of the stomach or small intestine. Moreover, in contrast to the distinct difference between the highly variable upper gastrointestinal tract microbiota and relatively uniform large bowel microbiota present within the lumen, there is a regional continuity present in mucosal microbial communities throughout the length of the equine gut.

## Supporting Information

S1 Fig Hierarchical clustering of luminal samples. Unweighted pair group method with arithmetic mean (UPGMA) of Bray-Curtis similarity indices between luminal microbiota detected in the dorsal stomach (DS), antral stomach (AS), jejunum (Jej), ileum (Ile), cecum (Cec), ventral colon (VC), and dorsal colon (DC) of nine healthy adult horses. Cophenetic correlation = 0.9516.
(TIF)

S2 Fig Hierarchical clustering of mucosal samples. Unweighted pair group method with arithmetic mean (UPGMA) of Bray-Curtis similarity indices between mucosal microbiota detected in the dorsal stomach (DS), antral stomach (AS), jejunum (Jej), ileum (Ile), cecum (Cec), ventral colon (VC), and dorsal colon (DC) of nine healthy adult horses. Cophenetic correlation = 0.8263.
(TIF)

S1 Table Summary statistics of coverage for all samples included in the analysis. (XLSX)

S2 Table Operational taxonomic units detected at greater than 0.01% mean relative abundance in luminal samples from any single region of the gastrointestinal tract. (XLSX)

S3 Table Operational taxonomic units detected at greater than 0.01% mean relative abundance in mucosal samples from any single region of the gastrointestinal tract. (XLSX)
