# Supplementary figures and images for: A Microbiological Map of the Healthy Equine Gastrointestinal Tract
Source: PLoS One. 2016 Nov 15;11(11):e0166523. doi: 10.1371/journal.pone.0166523 (PMC5112786; doi:10.1371/journal.pone.0166523)

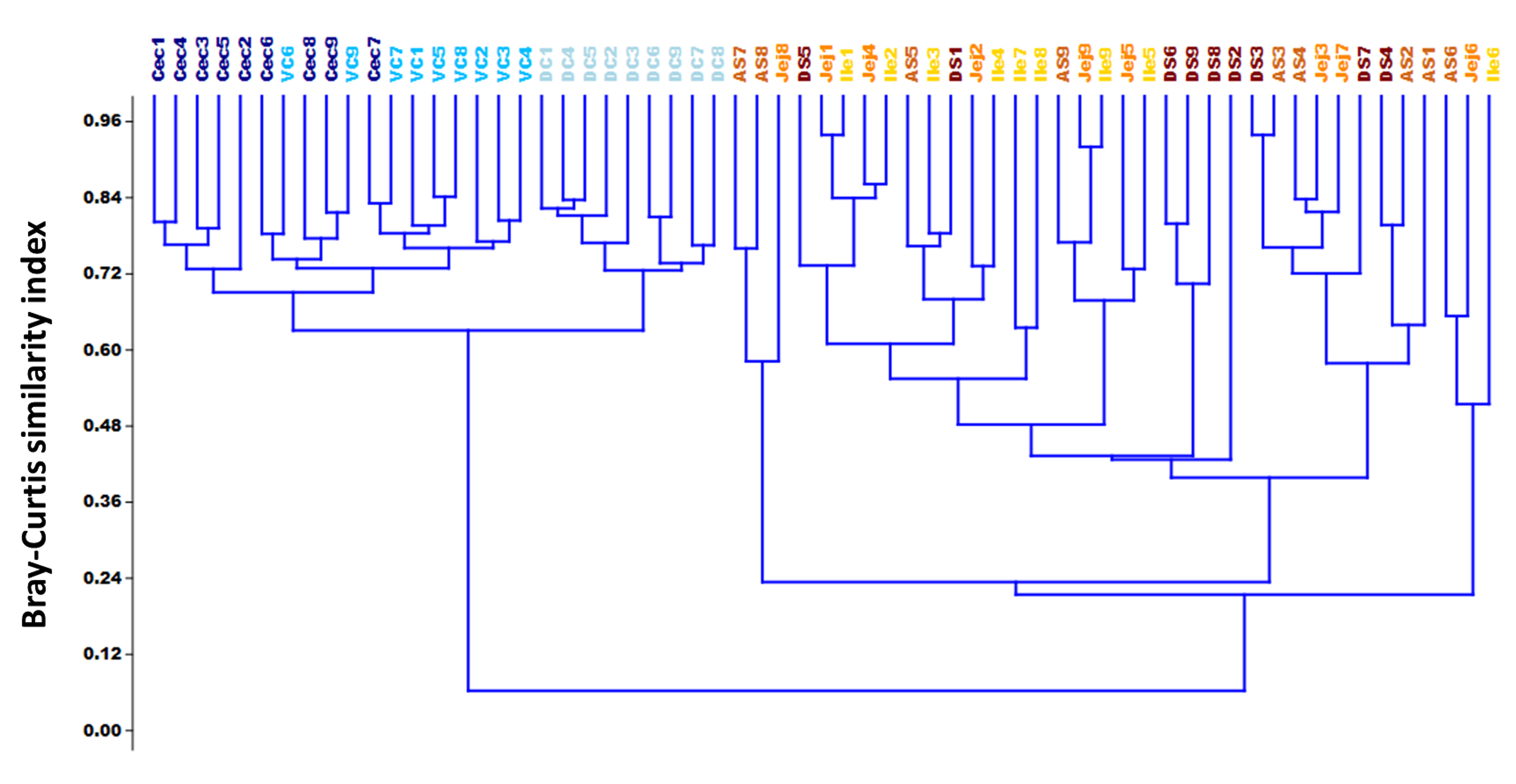

Supplement: S1 Fig — Unweighted pair group method with arithmetic mean (UPGMA) of Bray-Curtis similarity indices between luminal microbiota detected in the dorsal stomach (DS), antral stomach (AS), jejunum (Jej), ileum (Ile), cecum (Cec), ventral colon (VC), and dorsal colon (DC) of nine healthy adult horses. Cophenetic correlation = 0.9516. (TIF) [file pone.0166523.s001.tif]

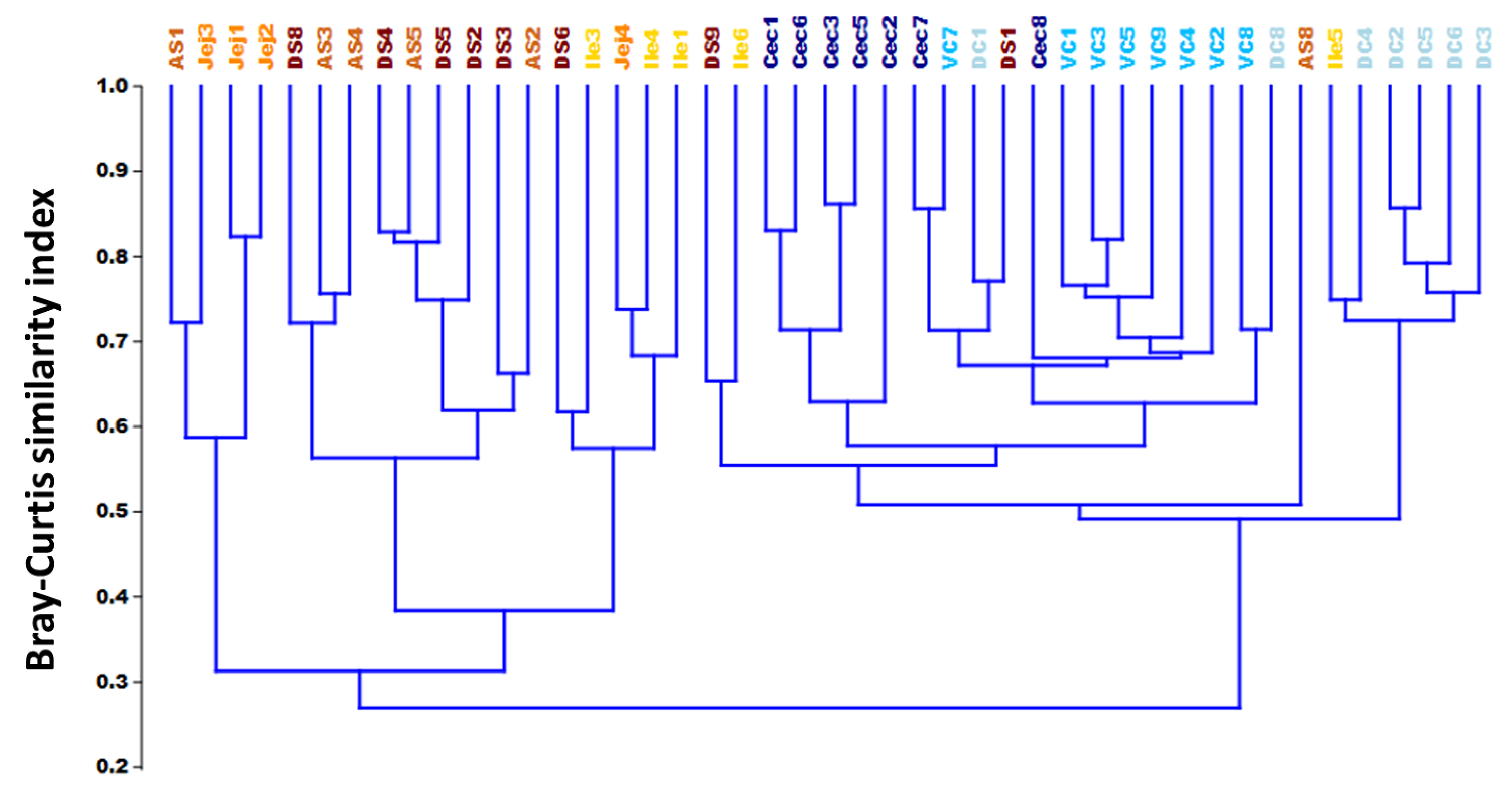

Supplement: S2 Fig — Unweighted pair group method with arithmetic mean (UPGMA) of Bray-Curtis similarity indices between mucosal microbiota detected in the dorsal stomach (DS), antral stomach (AS), jejunum (Jej), ileum (Ile), cecum (Cec), ventral colon (VC), and dorsal colon (DC) of nine healthy adult horses. Cophenetic correlation = 0.8263. (TIF) [file pone.0166523.s002.tif]
